# The Metropolitan Hospital Sunday Fund Meeting

**Published:** 1913-02-22

**Authors:** 


*To the Editor of* THE HOSPITAL.

### The Metropolitan Hospital Sunday Fund Meeting.

*To the Editor of* THE HOSPITAL.

SIR,—I drew attention at the Sunday Fund meeting to the fact that the Rev. Lionel Lewis, though posing as a friend of the Fund and anxious for its well-being, had not acted as such, and that deeds were of more value than professions of friendship. I pointed out that the collections in his church had fallen from £2 13s. 1d., which was the church's subscription when Mr. Lewis was appointed, to 3s. 3d. the year after he was appointed and to 8s. 11d. in 1912.

To this Mr. Lewis replied that his congregation was poor and that his parish contained many Jews and some Roman Catholics, and that the last incumbent was well off. But it would have been more to the point, and less misleading, if he had told the meeting that his own subscription of £1 1s. a year goes to the Anti-Vivisection Hospital, and that his congregation subscribed £1 1s. to that hospital. We may fairly assume that this was done at his instigation, because till he was appointed their collection went to the Sunday Fund, and his congregation certainly use the London Hospital and not the Anti-Vivisection.

Mr. Lewis "deplored that the gifts of the poor were ridiculed." What humbug! What he has much more reason to deplore is that his profession of friendship for the Fund and anxiety for its welfare have been exposed. It was his explanation for the reason of the miserable collection in his parish which was ridiculed as it deserved to be.—Yours truly,

SYDNEY HOLLAND.

